# Exposing the Value of Receiving and Giving Help across 12 European Countries: a Longitudinal Analysis of Self-Perceived Health

**DOI:** 10.21203/rs.3.rs-10795109/v1

**Published:** 2026-08-25

**Authors:** Hans Gevers

**Affiliations:** Estonian Business School, Department Research, Development & PhD Studies

**Keywords:** European Union, longitudinal, older people, self-perceived health, giving and receiving help

## Abstract

The act of receiving and giving help is commonly expected to improve older people’s health. In this article, this expectation is explored through a longitudinal analysis of a representative sample of 29,995 respondents aged 59 to 89 in 2011 from 12 European countries documented in the Survey of Health, Ageing and Retirement in Europe (SHARE) for the period 2011 to 2022. An unordered correlated random-effects (CRE), an ordered fixed-effects logistic (FEO), as well as an ordered random-effects logistic panel estimator (REO), all with longitudinal calibrated weights, are used to estimate the relationship between self-perceived health, receiving, and giving help. Additionally, a stereotype ordered logistic estimator (SO) is included to support the detected interaction effect between receiving and giving help. Overall, the study documents the positive impact on self-reported health status of income, doing activities and being satisfied with life, as well as the negative impact of age and having limitations. The Danish older people report the best health status, followed by the Swedes and the Swiss. Moreover, the study highlights that receiving help relates to worse self-perceived health, and that giving help relates to better self-perceived health. Above all, the study reveals the potential of the act of giving help to improve the self-perceived health status, in particular of those who receive help.

## Introduction

Active ageing is commonly regarded as a desirable phenomenon in our global society, given that it is expected to have a positive impact on health and subsequently a negative one on the care burden which societies face worldwide. A valuable social activity for an active life is helping out, which is, in the existing literature, primarily discussed in the context of voluntarism. This social activity of helping out is primarily expected to trigger beneficial effects on well-being and health. An overarching study of this association is provided by de Wit, Qu and Bekkers [1] through their ‘mega-analysis’ across 33 years and 22 European countries of six longitudinal panel surveys, SHARE included. They estimate that, through volunteering, a health advantage of, on average, more than 13 per cent is acquired. The health advantage tends to increase with age, and to be a priori larger for those with a worse state of health. On top of their fixed-effects regressions, first-difference estimators confirm the significant positive influence of continuing volunteering compared to stopping with it. This effect also results from the analysis of Kleiner et al. [2], who document the impact of volunteering on health through quality of life scores extracted from questionnaires and focus groups covering the older Swiss community. The scores are regressed with logistic estimators on variables covering age, gender, education, subsidy recipient, and impairment for both the states of stopped and continued volunteering. They find the odds of a decrease in quality of life to be higher in the stopped state, more precisely 75 per cent and 66 per cent higher for, respectively, self-esteem and autonomy. The study of Weziak-Bialowolska, Skiba, and Bialowolski [3] also supports the health advantage resulting from helping out. Based on SHARE-data from 15 European countries and with generalised estimating models, they document reciprocal longitudinal associations, of which the significant and positive associations of optimism and vitality with doing voluntary activity at least once a week are the most explicit. Other positive psychological well-being effects resulting from volunteering are illustrated by Huo, Miller, Kim, and Liu [4] through an autoregressive cross-lagged panel model based on the Health and Retirement Study Wave 2010 and 2014. Individuals aged 65 or older confirm significantly increased positive self-perceptions of ageing, and decreased negative perceptions when volunteering more than 100 hours. The effect appears not to significantly impact depressive symptoms, just as volunteering less than 100 hours overall. Volunteering at least 200 hours during the past year might even reduce the risk of deterioration or dying for adults with cardiovascular disease. Heisler et al. [5] conclude the latter based on the Health and Retirement Study in the United States with, among others, ordered logistic regressions. The importance of the frequency of volunteering is also illustrated by Lawton, Gramatki, Watt, and Fujiwara [6], who find that more volunteering results in higher well-being in the United Kingdom across a period of 20 years. With fixed-effects estimators they notice that self-rated health on a one-to-five scale increases with the volunteering frequency, i.e. from rarely (baseline) over several times a year (coefficient 0.098, p<.01) to at least once a week (coefficient 0.126, p<.01). Pavlova and Lühr [7] document an additional psychological impact of volunteering on social well-being with data from the 2012 European Social Survey covering 29 European countries. For their three dependent variables representing well-being, i.e. generalised trust, perceived social support, and loneliness, the positive impact of volunteering is significant at a 0.1 level. Significant independent variables at the same level are, among others, education, employment, income, gender, and self-reported general health. Additionally, the impact of offering help tends to vary geographically. Lakomy [8] distinguishes this variation between welfare regimes with panel fixed-effects regressions based on SHARE data from Waves 4 to 6. He finds that caregiving outside the household has no significant influence under any regime, but that providing care within the household significantly influences quality of life under all regimes except the social-democratic one, as found in Denmark or Sweden. In turn, under the latter regime, volunteering has a significant influence on the quality of life as well as in the Mediterranean countries, for example, Italy or Spain. Yet, in conservative countries like Austria, Belgium, France, or Germany, and post-communist countries, like Czech Republic, Estonia and Slovenia, the benefits of voluntarism for quality of life are expected to be limited. Additionally, Morawski, Okulicz-Kozaryn, and Strzelecka [9] find, also with SHARE data, that voluntary work positively correlates to quality of life. Their Kendall tau-b correlation coefficients reveal that intrinsic aspects like self-realisation relate to higher volunteering rates, not extrinsic ones like control. Meneghini and Colledani [10] notice the same dominant contribution of intrinsic motivation to satisfaction with life and well-being in their smaller sample of 133 Italians. They find a 40 per cent increase in life satisfaction allocatable to the commitment to volunteering. Besides improving life quality, those who help out others are also expected to live longer, an expectation which is mediated by prospective health [11]. Lastly, the impact of a health shock on self-perceived health is worth mentioning, given the more recent occurrence of COVID-19. The latter appears to undo the positive effect of volunteering, and might even trigger a negative effect of volunteering on well-being [12]. To conclude, the act of helping out is, besides factors like life satisfaction, health shocks or income, proven to influence well-being, self-perceived health included. Overall, past research indicates that helping behaviour facilitates healthy ageing.

On top of well-being, helping out relates to status. Three kinds of status are documented, i.e. job, financial, and social status. First, job status is known to influence volunteering as well as health. Hämäläinen et al. [13] find that retirement initiates more volunteering, in particular, for those who perceive having better health. Their results rely on fixed-effects panel regressions based on SHARE data from 2011 to 2018 of 19 European countries. Kalbarczyk and Łopaciuk-Gonczaryk [14] use the same dataset, limited to 2011 and 2015, for 14 European countries to conclude, based on a Generalised Structural Equation Model, that “active people remain active and inactive people remain so after retirement”. Besides acknowledging the relevance of retirement, the relationship between volunteering and employment is discussed. Strauss [15] scrutinises five waves of SHARE data from 2004 up to 2015 for 13 nationalities in the EU in the age category 50 years and older. He relies on multi-level random-effects as well as conditional fixed-effects logistic regressions to explore the impact of caregiving on volunteering. His research illustrates how caregivers tend to volunteer more than non-caregivers, as well as that cash-for-care expenditures stimulate both groups to volunteer, likely resulting from receiving compensation for the loss of income from paid work. Almeida-Meza, Di Gessa, Lacey, McMunn, and Xue [16] similarly find carers and former carers to volunteer more than non-carers across five waves of SHARE covering 16 European countries. Moreover, they note that the frequency of care positively relates to the frequency of volunteering. Second, on top of job status, the relationship between financial status and frequency of helping is documented. Hämäläinen et al. [17] explore Waves 4 to 8 of SHARE with a fixed-effects ordered logit model to find a positive relationship between the frequency of volunteering and deteriorating health as well as worsening financial condition. They find that a change to a worse financial condition increases the probability of not volunteering, while the inverse effect, i.e. a change to a better financial condition, appears to have no significant effect. Besides job status, financial status, as measured by income, influences the effect of frequency of participation in formal social activities on loneliness, being an aspect of mental health. Nissanholtz-Gannot and Peretz-Dayan [18] scrutinise this effect through hierarchical multiple regression models for 18 European countries based on Wave 6 of SHARE. Formal social activity is defined as participating in, e.g., charity or social organisations, and can be considered as a conditioned form of helping, as it mostly comes with private financial expenses. Nissanholtz-Gannot et al. find frequent formal social activity relevant for the lower income quintiles, as it significantly reduces the self-perceived loneliness scores. In a similar social context, Näsman et al. [19] discuss formal volunteering jointly with three other factors of civic engagement, e.g., political participation. Relying on data from the European Quality of Life Survey covering 32 European countries, their logistic regressions reveal significant relationships between formal volunteering and individual resources, i.e. education level, income, and health. They conclude that civic engagement, volunteering included, benefits from additional investment in the individual resources on top of the country-level social expenditures. As such, the individual as well as the national financial context is relevant for cultivating a culture of helping out. Lastly, the societal recognition or position a volunteer enjoys is of interest. Sánchez-García et al. [20] conclude, based on 29 countries covered in the European Social Survey (ESS), that people aged 70 years and older “who volunteer are 1.7 times more likely to report better health and life satisfaction”. Additionally, their multilevel regression models with fixed and random effects reveal a significant moderation effect of older people’s social status on volunteering. To conclude, the act of helping others is expected to occur in a context conditioned by the financial, work, and social status.

### Data

The longitudinal analysis in this study relies on data from SHARE [21, 22]. The latter is a recurring health survey that inquires European citizens about the effects of health, social, economic and environmental policies. The data used for this study covers Waves 4 to 9, and 12 countries in Europe, being Austria, Germany, Sweden, the Netherlands, Spain, Italy, France, Denmark, Switzerland, Belgium, Czech Republic, and Slovenia. [23–28]. The dataset based on the six SHARE waves is unbalanced on the panel level, i.e. on the level of an individual respondent, due to the fact that the same respondents from Wave 4 are taken forward up to Wave 9 without replacement. The latter is accounted for by integrating calibrated longitudinal weights in the estimators. Additionally, the panel time level comes with gaps resulting from the frequency of surveying; nevertheless, this is no cause for concern. Nonetheless, the dataset is well balanced based on gender, as on average 43.9 per cent of the respondents are female and 56.1 per cent are male. Worth mentioning is that it primarily regards retired respondents, given that 75.9 per cent report being retired in Wave 4 (2011), up to 87.9 per cent in Wave 9 (2022).

Besides the structure, the content of the dataset is highlighted. As Table 1 illustrates, the dataset consists of 16 variables, of which four are considered continuous. It consists of 109,546 observations for 29,995 respondents, each reporting maximally six times across the panel time horizon.

Table 1 reveals the one-to-five scale of the dependent variable self-perceived health, which is the health status from Excellent to Poor. The independent variables are primarily individual characteristics, except for the occurrence of COVID-19, the respondent’s unique number and year of survey. As such, the pandemic is specified with a binary variable, which is set to one when the year of survey is 2022. The responses of Wave 8, i.e. from 2020, are not marked as 70 per cent of the interviews for that wave were already conducted at the time of the lockdown [29]. The total time horizon covers Wave 4 for 2011, Wave 5 for 2013, Wave 6 for 2015, Wave 7 for 2017, Wave 8 for 2019/2020, and Wave 9 for 2021/2022. The respondent’s individual characteristics are reflected through 13 independent variables, of which two are of prior interest. First, the former are summarised. Age has a range from 59 to 100 and a mean of approx.74 years, while the respondents’ years of education range from zero to 30, with a mean of 10 years. Note that respondents are 50 to 89 years old in the starting year 2011. Total household income is stated, if required through conversion, in Euro and has a minimum of zero and a maximum of approx. 94,000 euros. On average, a household reports a total household income of 27,489 euros, which can consist of earnings from any kind of employment, pensions, benefits, rent, financial products, as well as those from other household members. Total household income is preferred as it reflects the financial power of the respondent’s household, which is assumed to be available for supporting the living conditions of the household as a whole, including the respondent. Given its relatively wide scale and extreme values, the income variable has been winsorized at the 5^th^ and 95^th^ percentiles, and subsequently standardised by subtracting the mean from each income and dividing the outcome by the standard deviation. Besides household income, the household type is included in three non-overlapping categories, namely “Single”, “Couple, both responding”, and “Multiple, at least 1 responding”. This variable results from regrouping the original variable with nine categories, of which the categories “Single”, with 37,717 observations, and “Couple, both responding”, with 62,737 observations, are preserved. The newly created category “Multiple, at least 1 responding” comprises the remaining 9,092 observations. Additionally, the variable Job situation indicates if the respondent is retired, employed or self-employed, unemployed, permanently sick or disabled, homemaker, or in another situation (e.g., studying or volunteering). The Job situation variable includes, as three other variables, a category “Not in universe”, which is not included in the output yet bundles the feedback of respondents refusing to answer, not knowing an answer, and the like. For Job situation, this category accounts for 1.6 per cent of the total number of observations. The Being limited binary indicator, also known as the Global Activity Limitation Indicator, reflects a respondent’s limitation in activities because of a health problem, and denotes “Not limited” or “Limited”. The Number of activities last year originally reflects whether the respondent did sports, reading, charity, and the like. It is regrouped to “None”, i.e. participated in no activities, and “At least 1”. It also includes the category “Not in universe”, which accounts for 1.6 per cent of the total number of observations. The variable Country holds the original SHARE country numbers for the 12 European countries in the dataset, being Austria, Germany, Sweden, the Netherlands, Spain, Italy, France, Denmark, Switzerland, Belgium, Czech Republic, and Slovenia. Lastly, the value of a helping hand is reflected in the covariates of interest Receiving help from, and Giving help to others. Both cover help from or to people inside or outside the household provided during the last 12 months, and originally specify an exact number of persons. Both have been converted to a variable with categories “None” and “At least 1”. The “Not in universe” category occurs in both the Receiving and Giving help variables, with, respectively, 7.6 per cent and 21.7 per cent of the total number of observations. In conclusion, the dataset variables are constructed to facilitate the regression analysis as well as to safeguard the integration of a maximum of known relevant determinants.

**Fig 1:**
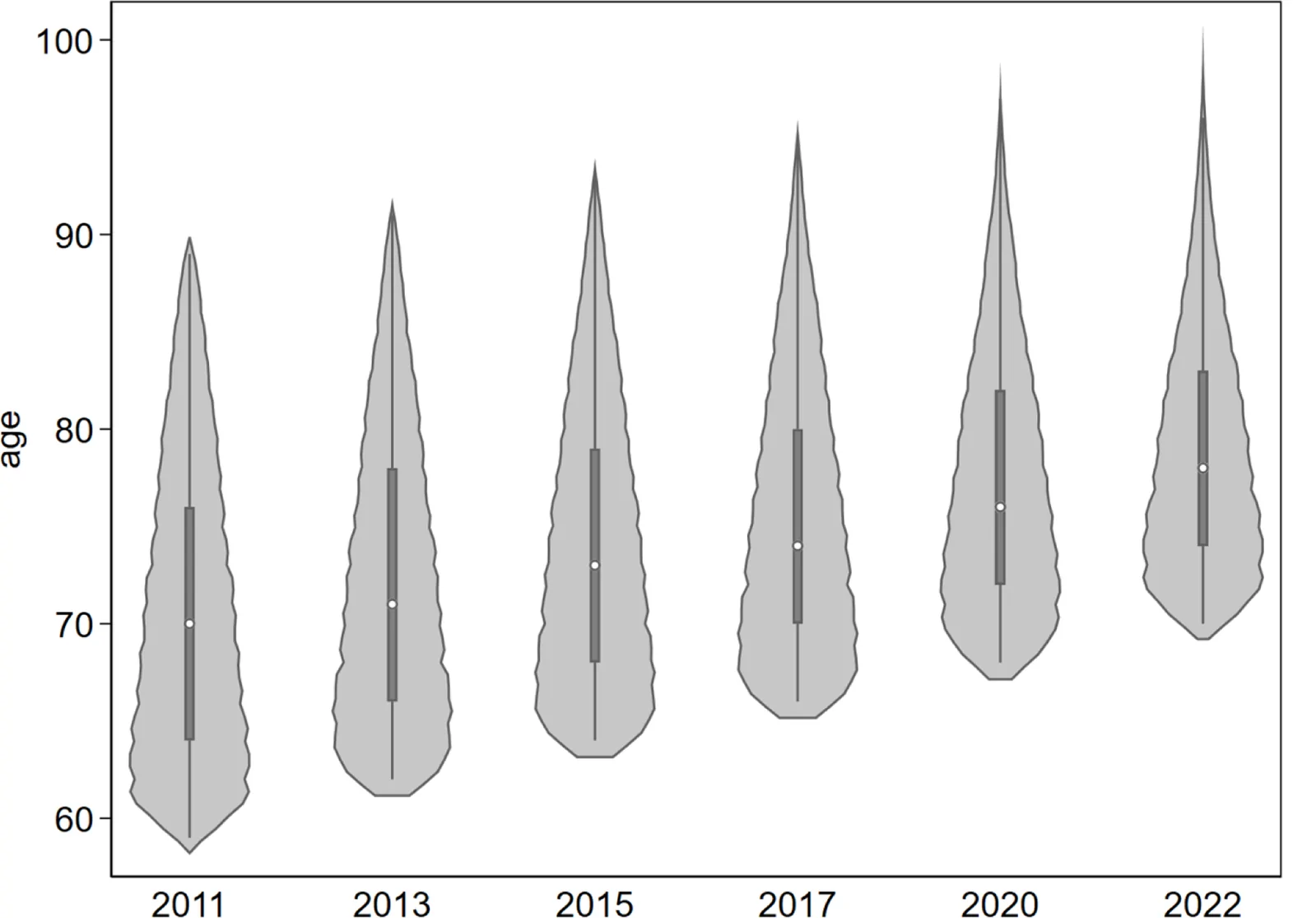
Age distributions per wave across countries from 2011 to 2022. Based on SHARE-data Wave 4 to 9.

**Fig 2:**
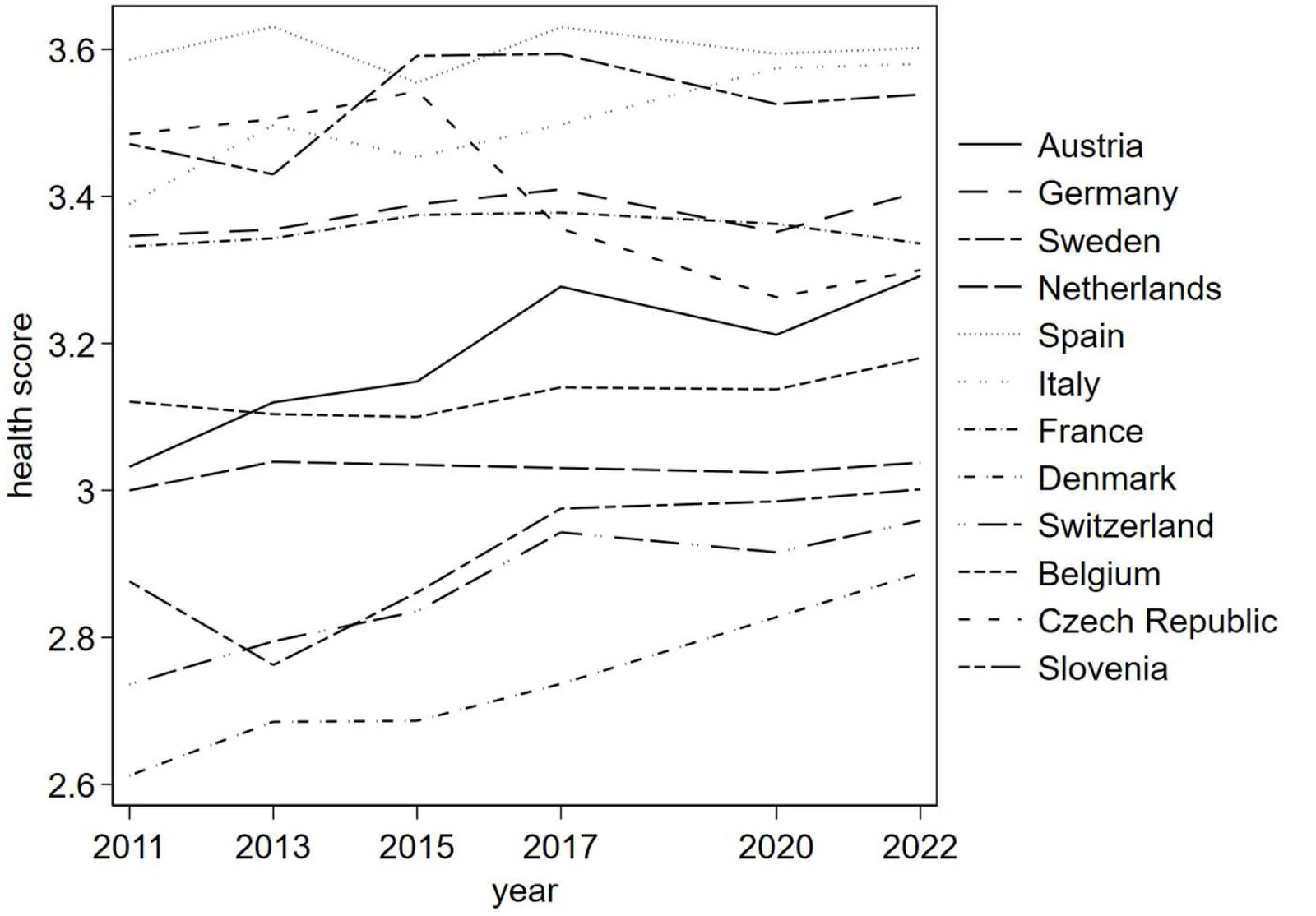
Average self-perceived health status per country per year

To visualise the time dimension of the dataset, two figures are included. Fig1 shows the age distribution of the respondents over time, while Fig2 plots the average self-perceived health score per country over time.

Fig1 shows how the age distribution of respondents in the dataset varies across the time horizon. It reflects how the respondents’ average age moves from 70 years old in 2011, closer to the age of 80 in 2022. Moreover, the plot visualises the ageing of the sample population. Fewer respondents appear to reach the higher ages.

Fig 2 reflects the average self-perceived health score across respondents per country per year. Denmark consistently notes the best, yet declining health scores across the time horizon, followed by Switzerland and Sweden. The Netherlands, Belgium and Austria cover more average scores. Among the low performers, the health status of the older people in the Czech Republic appears to have improved. France, Germany, Slovenia, and Italy complete the ranking. The lowest rank is for the older people in Spain, who consistently mark, on average, the lowest scores across the time horizon.

Besides the time dimension, the correlation structure of the variables is verified to ensure that the variables are compatible in regression analysis. Both the Pearson and Spearman Rank Order correlations are referred to. Table 2 illustrates the Pearson correlations.

Table 2 reveals that the Pearson coefficients are well below a 0.60 threshold. Additionally, the Spearman Rank correlations are calculated between all variables (see Table 14 in Appendix). Being limited appears strongly yet soundly related to self-perceived health, given the 0.533 correlation coefficient. Life satisfaction modestly correlates with the dependent variable by noting a −0.353 coefficient. On top, Total household income appears correlated with the Household type as well as with Receiving help, given their coefficients of, respectively, 0.352 and −0.358. Overall, the correlation tests do not suggest the exclusion of any of the selected variables for the regression analysis.

Two final characteristics of the dataset are worth mentioning. First, as commonly experienced with surveys, SHARE has issues of non-response and subsequently missing values. The SHARE data uses two approaches to handle these issues, i.e. the Hot-deck Imputations and Fully Conditional Specification approach. The former is used for negligible fractions of missing data, primarily of socio-demographic characteristics, and replaces the missing values with those of a similar recipient or donor. The latter handles the sizeable fractions of missing values, primarily in monetary variables. The imputation procedure is repeated five times, which results in five imputed datasets differing only in values, not structure. Completing a robustness test by repeating the regression analysis across the five imputed datasets is motivated by the fact that 12 of the 14 regressed variables, including the dependent variable, are referred to as being imputed with one of the two imputation procedures [29]. The completed robustness tests are not repeated in this version of the working paper and consultable in a previous version [30]. The tests reveal that the imputation procedures do not alter the conclusions documented in this paper. Second, the analysis of SHARE data across the targeted time horizon preferably integrates calibrated longitudinal weights, which are calculated based on the reweighing procedure advised by Pacifico [31]. This procedure reweighs the data according to the mortality per year, country, age and gender as provided by Eurostat. It aims to “compensate for both problems of unit nonresponse in the baseline and refreshment samples of each wave, and problems of attrition in the longitudinal samples of different waves” [32]. Both imputations and weights are expected to improve the longitudinal analysis.

## Methods

This study implements a brief descriptive as well as an extensive regression analysis. The former maps, per category, the self-perceived health per year, its transitions between years, and its relation to the two covariates of interest, being Receiving and Giving help. For the regression analysis, a set of estimators is programmed in Stata 19.5 Basic Edition. It regards the commonly used fixed-effects (FE) and random-effects panel estimators (RE), with a preference for the additional variants that can take the order of the dependent variable, i.e. self-perceived health status, into account. A first concern is the choice between the fixed-effects and random-effects estimators. As such, for the dataset used, the Breusch and Pagan Lagrange test suggests not to accept the null hypothesis that the unit-specific error term has zero variance (Chi Squared 15,517.97, p=.0). Thus, the use of a random effects estimator is most efficient. Yet, the Hausman specification test reveals that the differences between both estimators are significantly different from zero (Chi squared 7950.301, p=.0), and the Mundlak specification test shows that the unit-specific error terms (i.e. per respondent) are correlated with the covariates (Chi squared 7374.43, p=.0). The three test results combined point towards the use of an unordered random-effects estimator with the Mundlak correction, i.e. CRE. This correction implies adding the time averages of the explanatory variables per respondent, which subsequently allows estimating the unit-specific error terms. Applying the Mundlak reasoning to an ordered estimator is suggested by the work of Cullinan, Gannon, and Lyons [33] who use it for a pooled ordered probit model with standard of living on a one-to-six scale as the dependent variable. Nevertheless, implementing this reasoning goes beyond the scope of this study as it requires additional theoretical support. Besides the REO, the ordered fixed-effects logistic panel estimator or FEO of Baetschmann, Ballantyne, Staub, and Winkelmann [34] is documented for comparison. Lastly, as they differ, the underlying estimation methods of the estimators are summarised subsequently. The FE relies on the Ordinary Least Squares estimation method, while RE and CRE are implemented with the Generalised Least Squares method. The FEO uses the Blow-up and Cluster method, and the REO the Maximum Likelihood method. A priori, the CRE, FEO and REO are preferred. The latter are implemented with robust standard errors according to the estimation approach of Huber [35] and White [36]. Moreover, they integrate calibrated longitudinal weights specifically calculated for this study, and report coefficients or odds ceteris paribus.

### Descriptive analysis

To exploit the informativeness of the data, a descriptive analysis is documented of the dependent variable, i.e. self-perceived health, as well as of the two covariates of interest, i.e. Receiving and Giving help. Table 3 on the following page shows the distribution of older people’s reported health status across its five categories per year.

Table 3 illustrates that a category’s rank within years holds across the years. As such, the categories fluctuate around the following percentages across the time horizon: “Good” (approx. 40 per cent), “Fair” (approx. 29 per cent), “Very good” (approx. 15 per cent), “Poor” (approx. 11 per cent) and “Excellent” (approx. 5 per cent). On top, the health status moves gradually towards the middle category, as reflected by the combined decrease in 2022 of the categories “Poor” and “Excellent” of approximately three percentage points in favour of the category “Good”. Another interesting movement is how the respondents transition between categories over the years. Table 4 documents the transitions for the entire time horizon.

The transition probabilities in Table 4 on the main left diagonal indicate that the respondent most likely rates the same health score. Moreover, no large shifts to the extreme categories can be noted, as illustrated by the low percentages in the top right and bottom left corners. A respondent appears to move primarily to neighbouring categories. As such, with a 33.4 per cent likelihood, the respondent shifts from “Excellent” to the nearest lower category “Very good”, while, likewise, with a 38.7 per cent likelihood from “Very good” to “Good”. The transition from “Good” is also primarily downwards, given that a respondent moves 23.8 per cent of the time to the category “Fair”. The lowest but one category turns the trend as a 28.7 per cent likelihood applies to the shift from “Fair” to “Good”. The lowest category, “Poor”, notes a 34.3 per cent likelihood that a respondent shifts to the category “Fair”.

After the dependent variable, the two variables of interest are described and subsequently analysed. Table 5 on the following page describes the Receiving help variable according to the categories of self-perceived health.

Table 5 shows that, over time, a minimum of 50.1 per cent and a maximum of 88.9 per cent of the respondents report receiving no help, while a minimum of 11.1 per cent and a maximum of 49.9 per cent apply to those who report receiving help. These boundaries indicate that respondents dominantly receive no help across the time horizon. Another finding is that receiving help negatively relates to health status. Of those who report a poor health status, 43.7 per cent of all respondents across years report receiving help, while of those who report an excellent health status, merely 14.7 per cent receive help. This is no surprise as poor health suggests a need for additional help, and vice versa. Across categories and years, the majority of the older people consistently report not receiving help; however, across years, the cohort that receives help grows from 20.4 per cent in 2011 to 31.4 per cent in 2022. Overall, an increasing trend in receiving help arises from the data. To also reveal the trend in the variable Giving help, it is described in Table 6.

For Giving help, an inverse trend can be concluded as giving help positively relates to self-perceived health status. In total, 37.3 per cent of the older people across years who report excellent health actually give help to at least one other, while merely 10.8 per cent of those who report poor health provide a helping hand. Overall, a larger part of the population appears not to give help. Over time, a minimum of 55.7 per cent and a maximum of 91.7 per cent of the respondents report not giving help, while a minimum of 8.3 per cent and a maximum of 44.3 per cent applies to the givers of help. Moreover, a declining trend over the years suggests that fewer people give help. Potentially, the influence of the pandemic is relevant in the last two survey years, i.e. 2020 and 2022. To conclude, the descriptive analysis documents a diminishing trend of giving help and an increasing one of receiving help. In the subsequent section, the regression analysis will explore the relationships between self-perceived health and receiving as well as giving help in depth while accounting for the influence of other health determinants.

## Results

The value of a helping hand is estimated through five estimators, of which two are considered supportive for the choice of estimator, while three are regarded as valuable for interpretation. The coefficients with standard errors and the estimators’ specifications of the latter three estimators are listed in Table 7.

Table 7 shows the outcome of the CRE, FEO, and REO. To support the preference for these estimators the FE and RE are used as documented in Table 13 in Appendix. They are used for the Breusch and Pagan Lagrange test, the Hausman specification test, and the Mundlak specification test. The three tests suggest the RE being the most efficient, given that a Mundlak correction is implemented. The latter, i.e. CRE, is reported in column two and relies on a Generalised Least Squares estimation method which provides robust standard errors. It integrates longitudinal weights on top of the Mundlak means, which appear most relevant as supported by the zero p-value of the Mundlak test. The primary disadvantage of the CRE is that it is unable to take the order of the dependent variable, i.e. self-perceived health, into account. Nevertheless, it explains a considerable amount of variation (R-Squared 0.447). Moreover, it reports a non-zero panel-level deviation (0.439), which supports the use of a panel instead of a pooled estimator, as well as a non-zero variance due to random individual differences, i.e. the random effects (0.319, see Materials and Code availability). On top, the CRE confirms the theory of Yair Mundlak as its time-variant coefficients are the same as those of the FE, yet estimated with the more efficient random effects method. The results of Table 7 are discussed in the following paragraphs per variable. The odds ratio of the REO is referred to between brackets unless specified otherwise.

First, the results for the time-invariant variables Gender and Country, inestimable for the FEO, are highlighted. As such, older women appear to report a better health status (CRE coefficient −0.018, SE 0.007, p<.05) on average across years, yet the REO marks this effect as insignificant (1.037, SE 0.041, p>.05), implying that there is no notifiable difference, on average across years, in the health reporting of men and women. For the variable Country, merely the ORs of the more accurate REO are interpreted as they express the same as the coefficients of the CRE. As such, Danish older people consistently mark a better health status (0.748, SE 0.063, p<.001) than the Swedish, while the odds to report a less healthy status than the Swedish are two to three times higher for older Spanish (3.204, SE 0.248, p<.001), German (2.729, SE 0.219, p<.001), Slovenian (2.641, 0.228, p<.001), Czech (2.156, SE 0.165, p<.001), Italian (2.403, SE 0.182, p<.001), and French citizens (2.249, SE 0.156, p.<.001). The remaining countries report a more average health status, ranging incrementally from the Netherlands (1.213, SE 0.096, p<.05), over Belgium (1.292, 0.088, p<.001), to Austria (1.326, SE 0.092, p<.001). Switzerland reports a non-significant coefficient (0.920, SE 0.068, p>.05), implying that it does not (statistically) differ from Sweden regarding average self-perceived health status.

Second, the time-variant variables are interpreted. For four continuous variables, nonlinearity is tested through their squares. For age and its square, the CRE marks the main effect and square as significant, the FEO marks neither as significant, and the REO solely marks the main effect as significant (1.124, SE 0.039, p>.05). On top, the CRE and FEO find neither the years of education nor its squares to significantly impact the health status. The REO finds a significantly lower odds of reporting worse health status when years of education increases (0.950, SE 0.012, p<.001). All three estimators mark the total household income (0.803, SE 0.025, p<.001) as well as the life satisfaction level (0.707, SE 0.031, p<.001) as positive influencers of health. The significant square of income suggest that the effect of wealth on health is concave, i.e. a decreasing increase (1.048, SE 0.015, p<.01). For the remaining seven categorical variables, one appears explicitly not significant, i.e. the dummy for COVID-19 (0.990, SE 0.039, p>.05). The variables Households with a couple of which both are responding (1.163, 0.049, p<.001), as well as with multiple members of which at least one is responding (1.210, 0.077, p<.01), considerably increase the odds of reporting a less favourable health status compared to older people who live alone. This is rather surprising as it highlights a negative effect of partnership, not the expected health and economic benefit of partnership. Furthermore, the variable representing the Job situation marks three out of its five categories significant, i.e. the category Unemployed (1.289, SE 0.089, p<.001), Homemaker (4.199, SE 0.597, p<.001), and Other (e.g., volunteer or student) (1.249, SE 0.104, p<.01). The REO fails to detect the expected negative influence of being Permanently sick or disabled relative to the baseline (1.020, SE 0.152, p>.05), i.e. the older people who are employed, as confirmed by the CRE and FEO. Yet, the REO may capture this impact of inability through the variable Being limited in activities due to health problems, as it more drastically increases the expectation of reporting worse health (7.672, 0.241, p<.001) in comparison to the CRE and FEO. Across estimators, a positive influence is expected of participating in at least one activity (0.701, SE 0.027, p<.001). Regarding the two variables of interest, all three estimators consider that receiving help significantly relates to the health status. In particular, the FEO and REO reveal that receiving help from at least one other person implies a, respectively, 22.3 and 49.4 per cent increase in the odds of reporting worse health (FEO 1.223, SE 0.059, p<.001; REO 1.494, SE 0.063, p<.001). This is as expected given that worse health soundly implies a higher need for help. The positive effects of giving help also appear significant across estimators, i.e. as suggested by the CRE (coefficient −0.025, SE 0.008, p<.01), FEO (0.903, SE 0.043, p<.05), and REO (0.844, SE 0.032, p<.001). The interactions with Giving and Receiving help are considered not significant by the CRE and FEO, yet the REO considers this significant (0.822, SE 0.061, p<.01). In the previous versions of this paper, the interaction effect of giving help and receiving help is not supported due to integration of the Mundlak means in the REO [30]. Given that the latter approach requires additional theoretical support, it goes beyond the scope of this study. To additionally explore the findings of the REO, SOs are deployed. In the subsequent paragraphs, the significant interaction effect detected by the SOs is revealed for the survey years 2011 and 2015.

The importance of the interaction effect between Giving and Receiving help is supported by the cross-sectional SO. It is used as it does not impose the parallel regression assumption, which typically accompanies ordered logistic non-panel estimators. For now, theoretical support is lacking to verify this assumption for ordered random-effects logistic panel estimators, yet, precautionary, the SO is implemented to further explore the detected interaction effect. The SO is developed by Anderson [37] and like the REO, a maximum-likelihood estimator. Table 8 tabulates the findings as referred to in the previous version of this paper [30]. It relies on the equation as well as longitudinal calibrated weights as documented in Table 7, yet runs a single regression per survey year.

Table 8 shows that in two survey years, i.e. 2011 and 2015 the interaction effects are rated as significant. In the year 2017 the effect is rather significant, given a p-value of 0.061 (see Materials and Code availability). To obtain a better estimate for the significant years, the SOs are repeated with a limited sample and the cross-sectional sampling weights provided by SHARE instead of the longitudinal calibrated weights. The sample is limited to the time horizon 2011 and 2015, and excludes the Netherlands as it has no observations for the dependent variable in 2015. Table 9 reveals the findings.

Table 9 reports for both years the significant main effects of Receiving as well as Giving help, which confirm that those who receive help tend to report worse health, while those who give help report a better self-perceived health status. The interaction effects also appear significant in both years, implying that those who receive and give help tend to report a better self-perceived health status than those who merely receive help. To facilitate interpretation of the estimation results from Table 9, the derived probabilities are tabulated in Table 10 on the subsequent page. The table solely contrasts the probabilities for those who receive help yet do not give help, versus those who receive as well as give help.

Table 10 reveals that those who receive help and also give help are more likely to report a better self-perceived health status. For both years, the probabilities (columns three and five) are higher for the health categories Excellent, Very good, and Good for those who receive and give help in comparison to those who merely receive help. The probabilities to report the lower health status Fair or Poor are higher for those who merely receive help in comparison to those who receive and give help. To explore the effect further, the SO is rerun per year for each country separately. The results are revealed in Tables 11 and 12.

For both years, Tables 11 and 12 reveal that the interaction effect between Receiving and Giving help is not consistently significant across countries. For 2011, Sweden and Italy appear to report a significant interaction effect, while for 2015, only Austria reports this effect to be significant. Despite that the panel estimators do not acknowledge the relevance of this effect, it is worthy to take into account for two reasons. First, the effect is intuitively sound as it aligns with the positive influences of volunteering, which is extensively documented in the existing literature. Second, the variable Giving help is for a considerable part, i.e. 21.7 per cent of the total number of observations, not informative, i.e. resulting from respondents refusing to answer, not knowing an answer, and the like. Overall, the results suggest that giving help likely has a positive impact on the self-perceived health status.

## Discussion

This study elaborates on the positive effects of giving help, which is commonly explored in the context of volunteering. It documents the relationship between self-perceived health status and receiving as well as giving help for older people in 12 European countries based on the data of SHARE. In particular, the results of cross-sectional SOs confirm the relevance of giving help for the self-perceived health of older people who also receive help. This finding aligns with the commonly concluded positive effect of volunteering and identifies a focus point for future research [1, 4, 9, 38]. Overall, the study aims to exploit the informativeness of the order in the dependent variable and provides robust results across estimators. Nevertheless, despite its strengths, the study is exposed to weaknesses. Exploiting informativeness through the order of the dependent variable implies not exploiting the health information available in the original dataset. The latter is commonly done by constructing an index which combines a series of health-related indicators, e.g. an index reflecting the quality of life [9]. Similarly, the act of helping out is represented by a single indicator, while a combination of indicators is feasible. E.g. Lakomy [8] maps the effect of separate activities like volunteering, care-giving, club membership, and the like, on well-being. Furthermore, all estimators failed to capture the effect of COVID-19 while a certain influence, primarily on mental health, is expected [38, 39]. For now, the simplicity of this binary indicator is the sole explanation for its insignificance. Additionally, solely the interaction effect of receiving and giving help is documented while other moderation or mediation effects may also be informative. For example, the relationship between self-perceived health status and receiving as well as giving help is expected to be influenced by prospective health, social culture, status and engagement [10, 11, 16, 40]. Lastly, a variable of interest, i.e. Giving help, reports almost a quarter of its observations in a non-informative category. Nonetheless, the potential occurrence of the interaction effect between receiving and giving help found in this study remains remarkable. For future research, the exploration of the persistent effects of receiving and giving help in relation to health status is proposed in order to further value the act of helping.

## Conclusion

Active ageing reflects a lifestyle which is beneficial for the health of older people. An important aspect of this lifestyle is the act of helping and its contribution to health. This study documents the value of giving help, which may appear to be additionally beneficial for European older people who also receive help. It modestly suggests seizing the opportunity of stimulating older people who receive help to also give help. This stimulus can be provided by caretakers, policymakers, family, and the like, as it is expected to improve the self-perceived health status overall.

## Figures and Tables

**Table 1:** Summary statistics for the dependent, independent and panel level variables

	Mean	SD	Minimum	Maximum

Self-perceived health	-	-	1	5
Age	73.863	7.675	59	100
Years of education	10.351	4.509	0	30
Total household income	27,489.207	24,343.792	0.000	93,998.211
Life satisfaction	7.812	1.705	0	10
Gender	-	-	1	2
Household type	-	-	0	2
Job situation	-	-	0	6
Occurrence of COVID-19	-	-	0	1
Year of survey	-	-	2011	2022
Respondent’s unique number	-	-	1	29,995
Being limited	-	-	0	1
Receiving help from others	-	-	0	2
Giving help to others	-	-	0	2
Number of activities last year	-	-	0	2
Country	-	-	11	34

Number of observations: 109,546 SD = Standard Deviation

**Table 2:** Pearson correlations between the continuous variables

		1	2	3	4

1	Age	1.000	-	-	-
2	Years of education	−0.147	1.000	-	-
3	Total household income	−0.171	0.171	1.000	-
4	Life satisfaction	−0.046	0.076	0.187	1.000

**Table 3:** Self-perceived health distribution per category in per cent per year

	2011	2013	2015	2017	2020	2022

Excellent	6.828	6.144	5.467	4.993	5.005	4.595
Very good	16.433	15.777	15.575	14.168	15.173	14.161
Good	37.576	38.797	37.986	39.700	40.738	40.671
Fair	28.008	28.187	29.533	29.350	29.300	29.669
Poor	11.155	11.096	11.440	11.789	9.785	10.904

**Table 4:** Self-perceived health transition percentages for the period 2011 to 2022

	Excellent	Very good	Good	Fair	Poor

Excellent	36.650	33.410	22.720	6.080	1.140
Very good	10.330	39.810	38.690	9.250	1.910
Good	2.670	13.070	56.220	23.830	4.200
Fair	0.770	3.420	28.710	52.260	14.840
Poor	0.270	1.290	10.280	34.330	53.830
Total	5.380	15.090	39.320	29.100	11.110

**Table 5:** Proportions of receiving help from others relative to the five levels of self-perceived health

	2011	2013	2015	2017	2020	2022	Total

Excellent							
Received help from no other	0.877	0.889	0.821	0.816	0.806	0.808	0.853
Received help from at least 1 other	0.123	0.111	0.179	0.184	0.194	0.192	0.147
Very good							
Received help from no other	0.866	0.868	0.808	0.784	0.782	0.788	0.833
Received help from at least 1 other	0.134	0.132	0.192	0.216	0.218	0.212	0.167
Good							
Received help from no other	0.837	0.843	0.791	0.755	0.739	0.716	0.799
Received help from at least 1 other	0.163	0.157	0.209	0.245	0.261	0.284	0.201
Fair							
Received help from no other	0.753	0.747	0.669	0.675	0.643	0.645	0.706
Received help from at least 1 other	0.247	0.253	0.331	0.325	0.357	0.355	0.294
Poor							
Received help from no other	0.610	0.601	0.523	0.530	0.505	0.501	0.563
Received help from at least 1 other	0.390	0.399	0.477	0.470	0.495	0.499	0.437
Total							
Received help from no other	0.796	0.796	0.729	0.716	0.698	0.686	0.755
Received help from at least 1 other	0.204	0.204	0.271	0.284	0.302	0.314	0.245

**Table 6:** Proportions of giving help to others relative to the five levels of self-perceived health

	2011	2013	2015	2017	2020	2022	Total

Excellent							
Giving help to no other	0.661	0.606	0.640	0.557	0.584	0.660	0.627
Giving help to at least 1 other	0.339	0.394	0.360	0.443	0.416	0.340	0.373
Very good							
Giving help to no other	0.686	0.677	0.653	0.642	0.666	0.718	0.674
Giving help to at least 1 other	0.314	0.323	0.347	0.358	0.334	0.282	0.326
Good							
Giving help to no other	0.744	0.732	0.726	0.716	0.738	0.778	0.739
Giving help to at least 1 other	0.256	0.268	0.274	0.284	0.262	0.222	0.261
Fair							
Giving help to no other	0.785	0.790	0.809	0.803	0.818	0.850	0.806
Giving help to at least 1 other	0.215	0.210	0.191	0.197	0.182	0.150	0.194
Poor							
Giving help to no other	0.874	0.884	0.898	0.902	0.917	0.901	0.892
Giving help to at least 1 other	0.126	0.116	0.102	0.098	0.083	0.099	0.108
Total							
Giving help to no other	0.756	0.749	0.754	0.740	0.760	0.799	0.759
Giving help to at least 1 other	0.244	0.251	0.246	0.260	0.240	0.201	0.241

**Table 7:** Estimation results for unordered as well as ordered fixed-effects and random-effects panel estimators

	CRE	FEO	REO

Age	−0.045*** (0.008)	0.993 (0.051)	1.124*** (0.039)
Age squared	0.000*** (0.000)	1.001 (0.000)	1.000 (0.000)
Years of education	−0.003 (0.007)	0.977 (0.041)	0.950*** (0.012)
Years of education squared	0.000 (0.000)	1.001 (0.002)	0.999 (0.001)
Total household income	−0.043*** (0.007)	0.908* (0.035)	0.803*** (0.025)
Total household income squared	0.011*** (0.003)	1.028 (0.019)	1.048** (0.015)
Gender: Female	−0.018* (0.007)	- -	1.037 (0.041)
Occurrence of COVID-19	−0.004 (0.009)	1.042 (0.056)	0.990 (0.039)
Household: Couple, both responding	0.076*** (0.013)	1.269** (0.093)	1.163*** (0.049)
Household: Multiple, at least 1 responding	0.099*** (0.017)	1.274* (0.130)	1.210** (0.077)
Job situation: Retired	0.048** (0.016)	1.029 (0.096)	1.360 (0.264)
Job situation: Unemployed	0.013 (0.040)	1.031 (0.204)	1.289*** (0.089)
Job situation: Permanently sick or disabled	0.168*** (0.032)	1.539** (0.247)	1.020 (0.152)
Job situation: Homemaker	0.047* (0.021)	0.945 (0.111)	4.199*** (0.597)
Job situation: Other	0.007 (0.029)	0.829 (0.119)	1.249** (0.104)
Being limited	0.427*** (0.007)	4.122*** (0.150)	7.672*** (0.241)
Life satisfaction	−0.033*** (0.008)	0.858** (0.044)	0.707*** (0.031)
Life satisfaction squared	−0.002*** (0.001)	0.995 (0.004)	1.000 (0.003)
Number of activities last year: At least 1	−0.061*** (0.010)	0.863*** (0.039)	0.701*** (0.027)
Receiving help: At least from 1 other	0.085*** (0.008)	1.223*** (0.059)	1.494*** (0.063)
Giving help: At least to 1 other	−0.025** (0.008)	0.903* (0.043)	0.844*** (0.032)
Receiving help: At least from 1 other & Giving help: At least to 1 other	−0.021 (0.013)	0.956 (0.082)	0.822** (0.061)
Country: Austria	0.060** (0.019)	-	1.326*** (0.092)
Country: Germany	0.205*** (0.021)	-	2.729*** (0.219)
Country: The Netherlands	−0.001 (0.022)	-	1.213* (0.096)
Country: Spain	0.309*** (0.021)	-	3.204*** (0.248)
Country: Italy	0.213*** (0.021)	-	2.403*** (0.182)
Country: France	0.168*** (0.019)	-	2.249*** (0.156)
Country: Denmark	−0.099*** (0.023)	-	0.748*** (0.063)
Country: Switzerland	0.072*** (0.021)	-	0.920 (0.068)
Country: Belgium	0.017 (0.019)	-	1.292*** (0.088)
Country: Czech Republic	0.123*** (0.021)	-	2.156*** (0.165)
Country: Slovenia	0.213*** (0.022)	-	2.641*** (0.228)
Constant	0.397 (0.355)	-	-
Number of observations	109,546	130,636	109,528
Number of respondents	29,995	19,389	29,989
R-squared	0.447	0.1295	-
Panel-level standard deviation	0.439	-	2.107
Mundlak test Chi squared	6817.660	-	-
Mundlak test p-value	0	-	-
Fixed effects	Yes	Yes	Yes
Random effects	Yes	No	Yes
Calibrated longitudinal weights	Yes	Yes	Yes
Mundlak means	Yes	No	No
Ordered	No	Yes	Yes
Standard errors (in parentheses) robust	Yes	Yes	Yes
Estimation method	GLS	BUC	ML

Baselines: Gender = Male, Household = Single, Job situation = Employed or self-employed, Receiving as well as Giving help = None, Estimators: CRE = Correlated Random-Effects, FEO = Fixed-Effects Ordered, REO = Random-Effects Ordered Estimation methods: GLS = Generalized Least Squares, BUC = Blow-up and Cluster, ML = Maximum Likelihood Estimation output: CRE reports coefficients, the FEO and REO report odds R-Squared: within for FE, overall for RE and CRE, pseudo for FEO Number of observations for FEO is 86,544 without copies. Regression output relies on the first imputation provided by SHARE. Age, Years of education, Total household income and Life satisfaction are continuous variables. All others are categorical.

*** p<0.001,

** p<0.01,

* p<0.05

**Table 8:** Receiving and Giving help estimation results for SOs – full sample

	2011	2013	2015	2017	2020	2022

Receiving help: At least from 1 other	1.226*** (0.177)	1.071*** (0.197)	1.268*** (0.176)	0.763*** (0.232)	0.800*** (0.228)	0.829*** (0.221)
Giving help: At least to 1 other	−0.478** (0.154)	−0.812*** (0.185)	−0.717*** (0.193)	−0.711** (0.234)	−0.527* (0.238)	−0.666* (0.283)
Receiving help: At least from 1 other & Giving help: At least to 1 other	−1.056** (0.337)	−0.628 (0.411)	−1.042** (0.354)	0.795 (0.424)	−0.271 (0.462)	0.151 (0.507)
Number of observations	29,989	22,899	18,545	16,181	10,710	11,204

Baselines: Receiving as well as Giving help = None. Robust standard errors in parentheses. Calibrated longitudinal weights are integrated. Regression output relies on the first imputation provided by SHARE.

*** p<0.001,

** p<0.01,

* p<0.05

**Table 9:** Receiving and Giving help estimation results for SOs – limited sample

	2011	2015

Receiving help: At least from 1 other	1.087*** (0.108)	1.182*** (0.104)
Giving help: At least to 1 other	−0.387*** (0.096)	−0.666*** (0.106)
Receiving help: At least from 1 other & Giving help: At least to 1 other	−0.693*** (0.193)	−0.816*** (0.187)
Number of observations	28,019	18,549

Baselines: Receiving as well as Giving help = None. Robust standard errors in parentheses. SHARE cross-sectional sampling weights are integrated. 11 European countries are regressed, i.e. the Netherlands is excluded from the sample. Regression output relies on the first imputation provided by SHARE.

*** p<0.001,

** p<0.01,

* p<0.05

**Table 10:** Probabilities from SOs for Self-perceived Health Status given Receiving and optionally Giving help

Health	2011		2015	
	Not helped	Helped	Not helped	Helped

Excellent	0.0446 (0.00192)	0.0637 (0.00315)	0.0349 (0.00167)	0.0568 (0.00276)
Very good	0.133 (0.00333)	0.164 (0.00468)	0.118 (0.00327)	0.163 (0.00486)
Good	0.359 (0.00329)	0.377 (0.00336)	0.361 (0.00392)	0.394 (0.00423)
Fair	0.313 (0.00384)	0.284 (0.00481)	0.336 (0.00440)	0.291 (0.00533)
Poor	0.151 (0.00411)	0.111 (0.00497)	0.150 (0.00397)	0.0953 (0.00453)
Number of observations	28,019		18,549	

Not helped = Receiving help: At least from 1 other & Giving help: None, Helped = Receiving help: At least from 1 other & Giving help: At least to 1 other. The tabulated probabilities are derived from the estimation referred to in Table 9. Health is the self-perceived health status. Standard errors in parentheses.

**Table 11:** Coefficients from SOs run per Country for the interaction effect between Receiving and Giving help of 2011

Austria	Germany	Sweden	Spain	Italy	France	Denmark	Switzerland	Belgium	Czechia	Slovenia

−0.636 (0.627)	−1.195 (0.944)	−1.414* (0.721)	0.478 (0.946)	−1.922* (0.837)	−0.599 (0.523)	−0.004 (0.652)	0.327 (0.791)	−0.397 (0.532)	−0.708 (0.435)	−1.010 (0.930)
3,462	1,305	1,681	2,627	2,601	3,756	1,374	2,398	3,221	3,846	1,748

Coefficients of the stereotype logistic estimators run per Country according to the same estimation equation as used in Table 9. All countries report significant main effects for Receiving and Giving help, except Germany, Sweden, Italy, France, Denmark, Belgium, and Czech Republic, which report non-significant coefficients for the main effect of Giving help. Sweden reports a non-significant main effect of Receiving help. Robust standard errors in parentheses. SHARE cross-sectional sampling weights are integrated. The last row reports the number of observations.

*** p<0.001,

** p<0.01,

* p<0.05.

**Table 12:** Coefficients from SOs run per Country for the interaction effect between Receiving and Giving help of 2015

Austria	Germany	Sweden	Spain	Italy	France	Denmark	Switzerland	Belgium	Slovenia

−1.869*** (0.525)	−0.972 (0.924)	0.669 (0.746)	1.877 (1.555)	−1.021 (0.857)	−0.668 (0.583)	−1.210 (0.708)	−0.269 (0.584)	0.306 (0.547)	−1.770 (0.999)
2,086	771	1,202	1,897	1,766	2,103	1,040	1,708	2,200	1,214

Coefficients of the stereotype logistic estimators run per Country according to the same estimation equation as used in Table 9. All countries report significant main effects for Receiving and Giving help, except Spain, Italy, Denmark, and Slovenia, which report non-significant coefficients for the main effect of Giving help. The stereotype logistic estimator failed to converge for Czech Republic. Robust standard errors in parentheses. SHARE cross-sectional sampling weights are integrated. The last row reports the number of observations.

*** p<0.001,

** p<0.01,

* p<0.05.

## Materials and Code availability

To provide insight into the longitudinal analysis completed in this article, do- and log-files of Stata are available via https://github.com/hansgevers/thevalueofahelpinghand. Additionally, the illustrations included in this paper, as well as the graphs of the calibrated longitudinal weights versus the design weights per country for imputed dataset one, are available via the hyperlink.

## Appendix

**Table 13:** Estimation results for unordered fixed-effects and random-effects panel estimators

	FE	RE

Age	−0.045*** (0.007)	0.014* (0.006)
Age squared	0.000*** (0.000)	0.000 (0.000)
Years of education	−0.003 (0.007)	−0.006* (0.003)
Years of education squared	0.000 (0.000)	−0.001*** (0.000)
Total household income	−0.043*** (0.006)	−0.072*** (0.006)
Total household income squared	0.011*** (0.003)	0.013*** (0.003)
Gender: Female	-	−0.002 (0.007)
Occurrence of COVID-19	−0.004 (0.009)	−0.012 (0.007)
Household: Couple, both responding	0.076*** (0.012)	0.059*** (0.008)
Household: Multiple, at least 1 responding	0.099*** (0.016)	0.072*** (0.011)
Job situation: Retired	0.048*** (0.014)	0.107*** (0.013)
Job situation: Unemployed	0.013 (0.040)	0.081* (0.035)
Job situation: Permanently sick or disabled	0.168*** (0.029)	0.436*** (0.025)
Job situation: Homemaker	0.047* (0.019)	0.130*** (0.016)
Job situation: Other	0.007 (0.028)	0.071** (0.025)
Being limited	0.427*** (0.006)	0.661*** (0.005)
Life satisfaction	−0.033*** (0.008)	−0.060*** (0.007)
Life satisfaction squared	−0.002*** (0.001)	−0.003*** (0.000)
Participated in at least 1 activity last year	−0.061*** (0.009)	−0.127*** (0.008)
Receiving help from at least 1 other (main effect)	0.085*** (0.008)	0.130*** (0.007)
Giving help to at least 1 other (main effect)	−0.025** (0.008)	−0.067*** (0.007)
Receiving and Giving no help	0.000 (0.000)	0.000 (0.000)
Receiving no help and Giving help to at least 1 other	0.000 (0.000)	0.000 (0.000)
Receiving help from and Giving help to at least 1 other	−0.021 (0.014)	−0.045*** (0.013)
Country: Austria	-	0.105*** (0.018)
Country: Germany	-	0.330*** (0.022)
Country: The Netherlands	-	0.047* (0.020)
Country: Spain	-	0.349*** (0.019)
Country: Italy	-	0.267*** (0.019)
Country: France	-	0.255*** (0.017)
Country: Denmark	-	−0.107*** (0.021)
Country: Switzerland	-	−0.009 (0.019)
Country: Belgium	-	0.085*** (0.018)
Country: Czech Republic	-	0.280*** (0.018)
Country: Slovenia	-	0.309*** (0.020)
Constant	4.078*** (0.277)	2.361*** (0.225)
Number of observations	109,546	109,546
Number of respondents	29,995	29,995
R-squared	0.118	0.394
Panel-level standard deviation	-	-
Mundlak specification test Chi squared statistic	-	7374.43
Mundlak specification test p-value	-	0
Fixed effects	Yes	Yes
Random effects	No	Yes
Calibrated longitudinal weights	No	No
Mundlak means	No	No
Ordered	No	No
Standard errors (in parentheses) robust	No	No
Estimation method	OLS	GLS

Baselines: Gender = Male, Household = Single, Job situation = Employed or self-employed, Receiving as well as Giving help = None, Country = Sweden Estimators: FE = Fixed-Effects, RE = Random-Effects Estimation methods: OLS = Ordinary Least Squares, GLS = Generalized Least Squares Estimation output: FE and RE report odds R-Squared: within for FE, overall for RE Regression output relies on the first imputed dataset (Imp1). Age, Years of education, Total household income and Life satisfaction are continuous, all others are categorical.

*** p<.001,

** p<.01,

* p<.05

**Table 14:** Spearman Rank Order correlation coefficients for all 16 variables, continuous and categorical.

		1	2	3	4	5	6	7	8	9	10	11	12	13	14	15	16

1	Self-perceived health	1.000	-	-	-	-	-	-	-	-	-	-	-	-	-	-	-
2	Age	0.222	1.000	-	-	-	-	-	-	-	-	-	-	-	-	-	-
3	Years of education	−0.193	−0.157	1.000	-	-	-	-	-	-	-	-	-	-	-	-	-
4	Total household income	−0.248	−0.165	0.181	1.000	-	-	-	-	-	-	-	-	-	-	-	-
5	Gender	0.054	0.028	−0.089	−0.130	1.000	-	-	-	-	-	-	-	-	-	-	-
6	Household type	−0.104	−0.241	0.061	0.352	−0.246	1.000	-	-	-	-	-	-	-	-	-	-
7	Job situation	−0.011	−0.174	−0.117	0.001	0.177	0.059	1.000	-	-	-	-	-	-	-	-	-
8	Occurrence of COVID-19	0.012	0.225	0.022	0.054	0.017	−0.062	−0.056	1.000	-	-	-	-	-	-	-	-
9	Year of survey	0.024	0.352	0.038	−0.117	0.024	−0.095	−0.135	0.536	1.000	-	-	-	-	-	-	-
10	Respondent’s unique number	0.051	0.027	−0.071	−0.003	−0.011	0.060	0.057	0.013	0.003	1.000	-	-	-	-	-	-
11	Being limited	0.533	0.206	−0.083	−0.151	0.065	−0.108	−0.032	0.035	0.042	−0.022	1.000	-	-	-	-	-
12	Receiving help from others	0.160	0.203	−0.014	−0.358	0.082	−0.188	−0.057	−0.009	0.208	−0.102	0.178	1.000	-	-	-	-
13	Giving help to others	−0.082	−0.193	0.054	−0.090	−0.055	0.144	0.025	−0.165	−0.150	−0.046	−0.053	0.227	1.000	-	-	-
14	Life satisfaction	−0.353	−0.032	0.068	0.190	−0.047	0.128	−0.007	0.024	0.029	−0.070	−0.226	−0.075	0.051	1.000	-	-
15	Number of activities last year	−0.166	−0.062	0.250	0.159	0.013	−0.017	−0.225	−0.010	0.006	−0.151	−0.071	0.005	0.045	0.125	1.000	-
16	Country	0.026	−0.017	0.165	−0.200	0.017	−0.021	−0.101	−0.015	0.014	0.000	0.024	0.069	0.017	−0.101	0.039	1.000
